# Efficacy of radioactive iodine therapy with concomitant antithyroid drugs in Japanese patients with Graves’ disease: a retrospective observational study

**DOI:** 10.1186/s13104-025-07558-9

**Published:** 2025-11-12

**Authors:** Yoshinori Osaki, Erika Matsuda, Hiroshi Fukazawa, Rikako Nakajima, Takaaki Matsuda, Yuki Murayama, Yoko Sugano, Hitoshi Iwasaki, Motohiro Sekiya, Hitoshi Shimano

**Affiliations:** 1 Department of Endocrinology and Metabolism, Institute of Medicine, University of Tsukuba, 1-1-1 Tennodai, Tsukuba, 305- 8575 Ibaraki Japan; 2 Department of Health care Center Yurigaoka, Mito Chuo Hospital, 1136-1 Rokutanda-chou, Mito, 310-0015 Ibaraki Japan; 3 Department of Endocrinology and Metabolism, University of Tsukuba Hospital, 2-1-1 Amakubo, Tsukuba, 305-8576 Ibaraki Japan; 4 Tsukuba Clinical Research and Development Organization (T-CReDO), University of Tsukuba, 2-1-1 Amakubo, Tsukuba, 305-8576 Ibaraki Japan

**Keywords:** Graves’ disease, ^131^I therapy, Radioactive iodine therapy, Antithyroid drugs

## Abstract

**Objective:**

Radioactive iodine therapy (RIT) for Graves’ disease (GD) often involves antithyroid drugs (ATDs) withdrawal and the exacerbation of thyrotoxicosis. This study investigated the efficacy of RIT with concomitant ATDs (CATD) versus temporary ATDs withdrawal in Japanese patients with GD, a population with high iodine intake according to global standards.

**Results:**

This retrospective observational study included 179 patients with GD who visited the University of Tsukuba Hospital. Propensity score matching (balancing age, sex, thyroid weight, free T4) created comparable groups of 15 patients each: CATD(-) withdrawing ATDs and CATD(+) continuing ATDs during RIT. Cure was defined as hypothyroidism or euthyroidism achieved without ATDs or potassium iodide. Post-matching, the CATD(+) group received significantly higher baseline ATD doses (median 600 vs. 200 mg/day propylthiouracil-equivalent; *p* < 0.008). The cure rate within 12 months after RIT was significantly higher in the CATD(-) group than in the CATD(+) group (*n* = 12 [80.0%] vs. *n* = 4 [26.7%]; *p* = 0.005). CATD may be associated with a lower cure rate within 12 months compared with standard RIT involving temporary ATD discontinuation. We suggest further prospective studies to confirm the efficacy of radioactive iodine therapy with concomitant antithyroid drugs in high iodine intake country.

**Supplementary Information:**

The online version contains supplementary material available at 10.1186/s13104-025-07558-9.

## Introduction

Graves’ disease (GD) is an autoimmune disorder characterized by hyperthyroidism, goiter, and ophthalmopathy and is common in women of childbearing age [1]. Radioactive iodine therapy (RIT) has been used as an effective and safe treatment option for patients with GD since first reported in the 1940s [2, 3], as the incidence of severe adverse events following RIT is usually low [4]. Hyperthyroidism rapidly improves after RIT administration [5]. However, several studies have reported transient exacerbations of hyperthyroidism early after RIT, particularly in patients who discontinued antithyroid drugs (ATDs) before RIT [6, 7]. A literature review indicates that worsening of the thyroid function occurs in approximately 10% of patients treated with radioactive iodine, with thyroid storm reported in about 0.3% of cases [8–12]. Administering RIT with concomitant ATDs in patients with hyperthyroidism may prevent post-RIT exacerbations of thyrotoxicosis or thyroid storms, which can occur following ATDs withdrawal [13–15]. The impact of concomitant ATDs on RIT outcomes has been investigated in several studies, with some reporting a reduced therapeutic effect [13, 15], while others found no significant difference [14, 15]. However, no similar studies have been reported from Japan, a country with exceptionally high iodine intake according to global standards [16]. Therefore, we conducted this retrospective study to investigate the efficacy of RIT concomitant with ATDs in Japanese patients with GD.

## Materials and methods

### Participants and study design

**Fig. 1:**
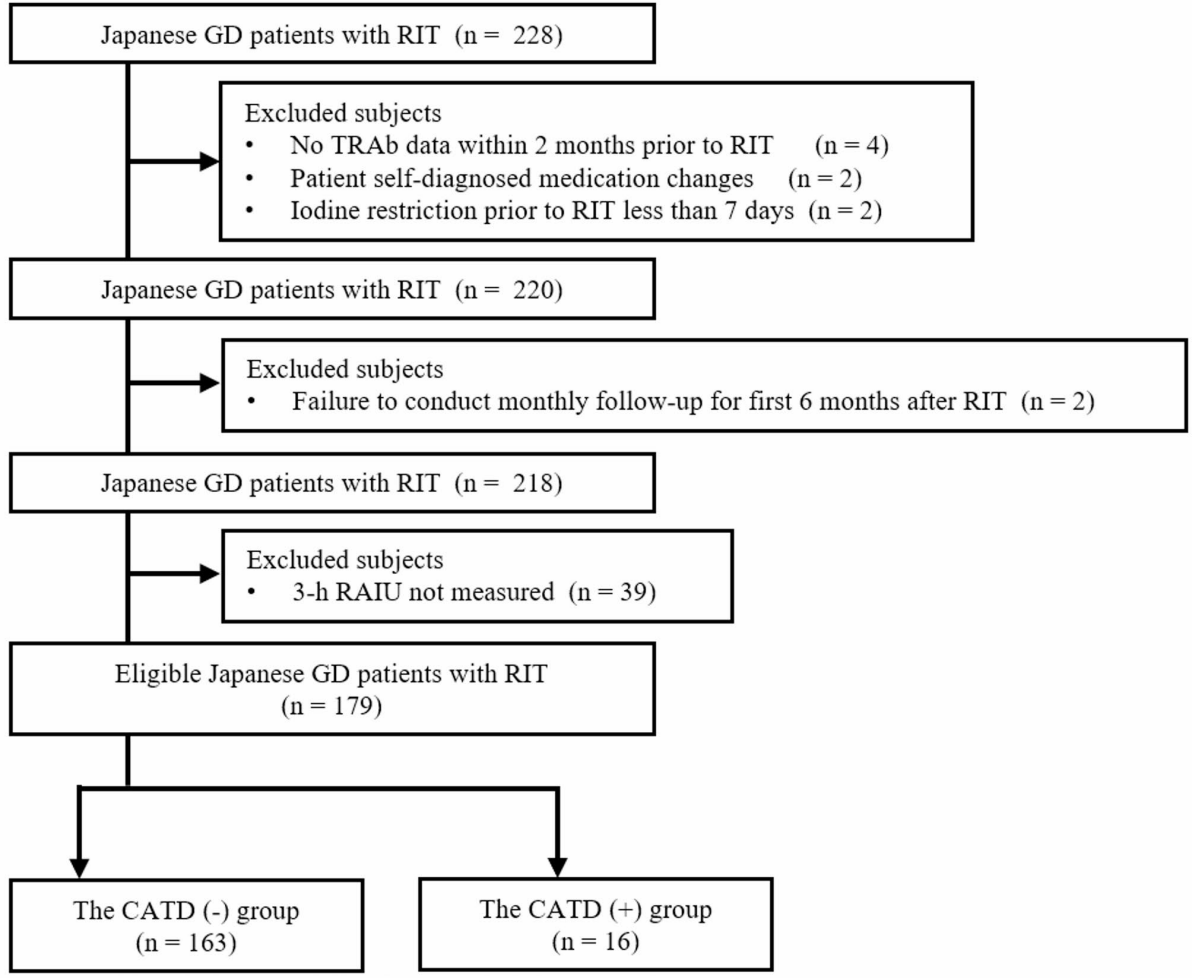
Participant inclusion process. Abbreviations: GD, Graves’ disease; RIT, radioactive iodine treatment; TRAb, TSH receptor antibody; RAIU, radioactive iodine uptake; CATD, RIT with concomitant antithyroid drugs

The participants were 228 Japanese patients with GD who underwent RIT between January 2013 and March 2024 at the University of Tsukuba Hospital. GD was diagnosed according to the 2013 Thyroid Disease Diagnostic Guidelines of the Japanese Thyroid Society [17]. Participant inclusion process was shown in Fig. 1. Finally, 179 eligible patients were included in this study and divided into two groups as follows: the RIT with non-concomitant ATDs group (CATD(-) group), in which ATDs were temporarily discontinued during the radioactive iodine uptake (RAIU) and RIT periods; and the RIT with concomitant ATDs group (CATD(+) group), in which ATDs were continued at either the same dose or at half the pre-RIT dose during the RAIU and RIT periods. The CATD(-) and CATD(+) groups consisted of 163 and 16 patients, respectively. CATD(+) group patients had either a current or past history of heart failure classified as New York Heart Association (NYHA) class II or higher; a history of thyroid storm; or severe symptoms such as fatigue, palpitations, and shortness of breath that significantly impaired their ability to carry out normal daily activities owing to hyperthyroidism. In addition, patients in the CATD(+) group strongly desired to avoid exacerbation of thyrotoxicosis due to discontinuation of ATDs during RIT. In this study, we defined the cure of GD post-RIT as meeting at least one of the following criteria in patients requiring neither ATDs nor potassium iodide (KI): (a) hypothyroidism or subclinical hypothyroidism; or (b) euthyroidism. As a methimazole (MMI) to propylthiouracil (PTU) dose ratio of 20:1 is advocated based on drug efficacy [18], the MMI dose was converted to the equivalent PTU dose and calculated as the “ATD dose” to analyze the effect of ATD dose, including both PTU and MMI, on RIT outcomes.

### Methods and ^131^I therapy

Serum free-T3 (FT3), Serum free-T4 (FT4), TSH, and TRAb levels were measured using electrochemiluminescence immunoassays (ECLusys FT3, FT4, TSH, and TRAb; Roche Diagnostics, Basel, Switzerland). The reference ranges at our institution were as follows: FT3, 2.3–4.0 pg/mL; FT4, 0.9–1.7 ng/dL; TSH, 0.5–5.0 µIU/mL; and TRAb, < 2.0 IU/L. Thyroid weight (TW) was measured as previously described [19]. Maximum width (W), maximum thickness (T), and maximum length (L) were measured in both the right (r) and left (l) thyroid lobes. TW was calculated using the following equation: TW = (rW × rT × rL + lW × lT × lL) × 0.70 [19]. For RAIU measurements and RIT, each patient restricted oral iodine intake and discontinued potassium iodide for more than 3 days before RAIU and for more than 7 days before RIT. In the CATD(-) group, ATDs were withdrawn 3 days before RAIU and 7 days before RIT. In the CATD(+) group, ATDs were continued at the same dose or at half the pre-RIT dose from 3 days before RAIU and 7 days before RIT. Discontinuation or reduction of ATDs, discontinuation of KI, and restriction of oral iodine intake were continued for 3 days after RIT. RAIU measurements were obtained 3 h after oral administration of ^123^I. The estimated 24-h RAIU values were obtained from the measured 3-h RAIU values using the following equation: estimated 24-h RAIU = 5.9 + 41.5 × log_10_ (actual 3-h RAIU) [20]. The therapeutic dose of ^131^I was calculated based on the estimated 24-h RAIU and TW. The calculated amount was obtained using the following equation: ^131^I dose (mCi) = 10 × (TW [g]) × (80–200 [µCi/g]) / (24-h RAIU [%]). For patients with normal thyroid function, 80–120 µCi/g was used as a guide; for hypothyroidism, 140–200 µCi/g was used. In patients with a calculated ^131^I dose exceeding 13 mCi, those who developed agranulocytosis due to ATDs, and those who desired a rapid and definitive cure with a single RIT, a fixed dose of 13 mCi was administered to avoid additional RIT. Patients underwent monthly blood tests and examinations for at least the first 6 months after RIT and were then followed up every 1 to 2 months until their GD was confirmed as cured.

### Statistical analysis

The data are expressed as medians and interquartile ranges for continuous variables and as numbers or percentages for categorical variables. For comparisons between two groups, the Mann–Whitney U test was used for continuous variables, and Fisher’s exact test was employed for categorical variables. To assess differences in the RIT cure rate between the CATD(-) and CATD(+) groups, a propensity score matching analysis was conducted [21]. The propensity score was calculated using logistic regression analysis with CATD group category as the dependent variable and age, sex, TW, and FT4 as covariates. These four covariates have been reported to be associated with RIT failure [22–25]. One-to-one nearest neighbor matching was used to match each patient in the CATD(+) group to a patient in the CATD(-) group with the closest propensity score. A caliper of 0.25 of the standard deviation of the propensity score in all subjects was applied to prevent poor matching [26]. For all tests, *p* < 0.05 was considered statistically significant. All statistical analyses were performed using SPSS version 28 (SPSS Inc., Chicago, IL, USA).

## Results

### Characteristics of the CATD(-) group and the CATD(+) group before matching

The characteristics of the CATD(-) group (*n* = 163) and the CATD(+) group (*n* = 16) before propensity score matching are shown in Table 1. The TRAb values, TW, and ATD dose were significantly lower in the CATD(-) group than in the CATD(+) group (5.3 IU/L [2.5–12.8] vs. 27.0 IU/L [4.8–38.6], *p* = 0.003; 32.6 g [24.5–45.8] vs. 72.2 g [47.5–78.4], *p* < 0.001; 200 mg/day [50–300] vs. 550 mg/day [400–600], *p* < 0.001, respectively). Conversely, there were no significant differences in 3-h RAIU values or the ^131^I dose between the two groups. The lack of difference in the ^131^I dose may be explained by the fact that many patients desired a rapid and definitive cure of GD with a single RIT owing to complications from other diseases, long travel distances, or long examination wait times, and thus received a fixed dose of 13 mCi of ^131^I.

**Table 1:** Characteristics of the CATD(-)and CATD(+) groups before matching

	CATD(-) group	CATD(+) group	*p*
**n**	163	16	-
Female, n (%)	131 (80.4)	10 (62.5)	0.112
Age (year)	45 (33–54)	40 (29–58)	0.664
Smokers, n (%)	30 (18.4)	2 (12.5)	0.741
TSH (µIU/mL)	0.156 (0-2.118)	0.011 (0-2.400)	0.362
FT4 (ng/dL)	1.13 (0.97–1.52)	1.10 (0.73–1.66)	0.549
FT3 (pg/mL)	3.2 (2.8-4.0)	3.9 (2.4–5.5)	0.540
TRAb (IU/L)	5.3 (2.5–12.8)	27.0 (4.8–38.6)	**0.003**
TW (g)	32.6 (24.5–45.8)	72.2 (47.5–78.4)	**< 0.001**
*MMI recipients, n (%)	93 (57.1)	10 (62.5)	0.794
*PTU recipients, n (%)	31 (19.0)	6 (37.5)	0.104
ATD dose (mg/day)	200 (50–300)	550 (400–600)	**< 0.001**
KI (mg/day)	0 (0–50)	50 (0-100)	0.182
Iodine Restriction before RIT (day)	7 (7–11)	7 (7–9)	0.973
3-h RAIU (%)	43.5 (30.2–58.9)	42.8 (31.3–54.4)	0.477
^131^I dose (mCi)	13 (13–13)	13 (13–13)	0.105

Continuous variables are shown as medians (interquartile range). Categorical variables are presented as numbers (percentages)

* MMI and PTU recipients indicate the number of recipients of MMI or PTU before RIT, respectively

Abbreviations: RIT, radioactive iodine treatment; CATD, RIT with concomitant antithyroid drugs; FT4, free T4; FT3, free T3; TRAb, TSH receptor antibody; TW, thyroid weight; MMI, methimazole; PTU, propylthiouracil; ATD, antithyroid drug; KI, potassium iodide; RAIU, radioactive iodine uptake

### Characteristics of the CATD(-) group and the CATD(+) group after matching

The characteristics of the CATD(-) and CATD(+) groups after propensity score matching are shown in Table 2. Fifteen patients in each group were matched using one-to-one nearest neighbor matching. No significant differences were observed in age, sex, TW, or FT4 levels between the two groups. However, the number of smokers was significantly higher, and the ATD dose was significantly lower, in the CATD(-) group than in the CATD(+) group (8 [53.3%] vs. 2 [13.3%], *p* = 0.025; 200 mg/day [50–400] vs. 600 mg/day [400–600], *p* < 0.008, respectively). On the other hand, there were no significant differences in the 3-h RAIU values or the ^131^I dose. Table 2 shows the patient background factors used to determine inclusion in the CATD(+) group. None of the three patients in the CATD(-) group with these factors requested RIT with concomitant ATDs. The characteristics of the entire CATD(-) group and the CATD(+) MMI or PTU recipients group after matching are shown in Supplementary Tables 1 and 2.

**Table 2:** Characteristics of the CATD(-)and CATD(+) groups after matching

	CATD(-) group	CATD(+) group	*p*
**n**	15	15	-
Female, n (%)	5 (33.3)	10 (66.7)	0.072
Age (year)	44 (36–49)	40 (28–60)	0.819
Smokers, n (%)	8 (53.3)	2 (13.3)	**0.025**
TSH (µIU/mL)	0.008 (0-1.010)	0 (0-0.568)	0.475
FT4 (ng/dL)	1.22 (0.99–1.87)	1.10 (0.71–1.69)	0.290
FT3 (pg/mL)	3.6 (3.0-4.9)	4.1 (2.5–5.5)	0.917
TRAb (IU/L)	6.9 (3.7–19.5)	31.3 (3.9–39.2)	0.089
TW (g)	62.6 (38.5–78.8)	70.6 (41.5–75.4)	0.319
*MMI recipients, n (%)	10 (66.7)	9 (60.0)	0.500
*PTU recipients, n (%)	2 (13.3)	6 (40.0)	0.107
ATD dose (mg/day)	200 (50–400)	600 (400–600)	**0.008**
KI (mg/day)	0 (0–50)	50 (0-100)	0.396
Iodine Restriction before RIT (day)	8 (7–11)	8 (7–9)	0.777
3-h RAIU (%)	44.2 (30.3–53.0)	42.4 (30.1–56.7)	0.604
^131^I dose (mCi)	13 (13–13)	13 (13–13)	1.00
**Patient’s Background Factors**			
**Coexistence or history of heart failure, n (%)	1 (6.7)	6 (40.0)	0.080
History of thyroid storm, n (%)	1 (6.7)	3 (20.0)	0.598
***Severe symptoms due to hyperthyroidism, n (%)	1 (6.7)	6 (40.0)	0.080

Continuous variables are shown as medians (interquartile range). Categorical variables are presented as numbers (percentages)

* MMI and PTU recipients indicate the number of recipients of MMI or PTU before RIT, respectively

** Coexistence or history of heart failure classified as New York Heart Association (NYHA) class II or higher

*** Severe symptoms such as fatigue, palpitations, and shortness of breath that significantly impaired their ability to carry out normal daily activities owing to hyperthyroidism

Abbreviations: RIT, radioactive iodine treatment; CATD, RIT with concomitant antithyroid drugs; FT4, free T4; FT3, free T3; TRAb, TSH receptor antibody; TW, thyroid weight; MMI, methimazole; PTU, propylthiouracil; ATD, antithyroid drug; KI, potassium iodide; RAIU, radioactive iodine uptake

### Changes of thyroid hormone levels in the period before and 2 weeks after RIT

**Fig. 2:**
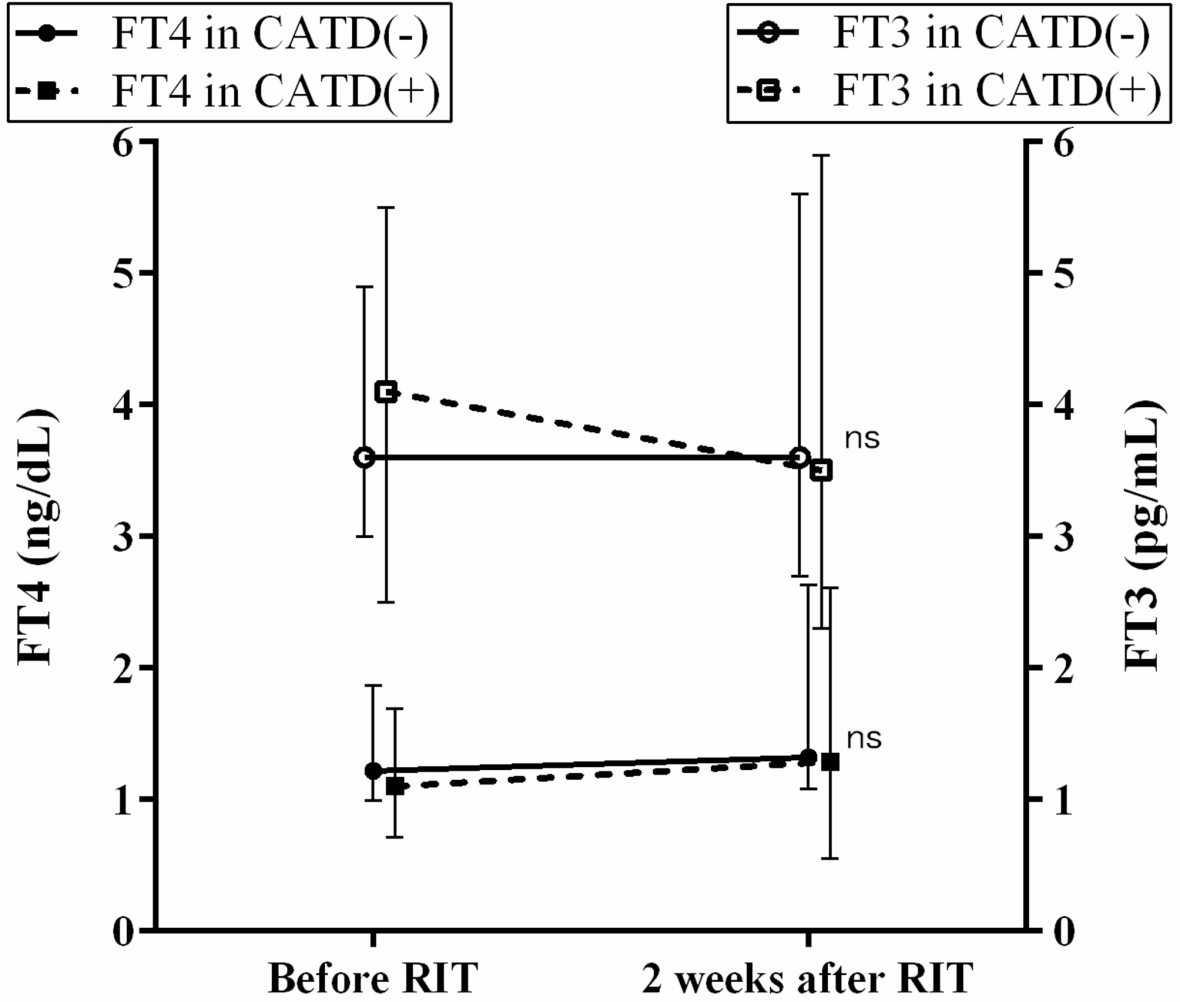
Thyroid hormone level changes before and 2 weeks after RIT. Graphs display median FT4 values (black circles or squares) and FT3 values (white circles or squares), with whiskers indicating the 25th and 75th percentiles

As shown in Fig. 2, no significant differences were observed in FT4 or FT3 values 2 weeks after RIT between the CATD(-) and CATD(+) groups (FT4: 1.32 ng/dL [1.08–2.63] vs. 1.29 ng/dL [0.55–2.61], *p* = 0.2169; FT3: 3.6 pg/mL [2.7–5.6] vs. 3.5 pg/mL [2.3–5.9], *p* = 0.9105). There was also no significant difference in the time from RIT to the first post-RIT blood test between the CATD(-) and CATD(+) groups (15 days [11–18] vs. 11 days [11–18], *p* = 0.849).

### Outcomes of RIT between the CATD(-) group and the CATD(+) group

The cure rate and time to cure after RIT in the CATD(-) and CATD(+) groups are shown in Table 3. The cure rates within 6 months and 12 months were significantly higher in the CATD(-) group than in the CATD(+) group (within 6 months: 12 [80.0%] vs. 1 [6.7%], *p* < 0.001; within 12 months: 12 [80.0%] vs. 4 [26.7%], *p* = 0.005). For patients not cured within 6–12 months after the first RIT, a second RIT was performed with patient consent. As a result, the proportion of patients requiring a second RIT was significantly lower in the CATD(-) group than in the CATD(+) group (1 [6.7%] vs. 9 [60.0%], *p* = 0.003). The cure rate in the entire CATD(-) group and the CATD(+) MMI or PTU recipients group after matching is shown in Supplementary Tables 3 and 4. The cure rate within 6 months was significantly lower in the CATD(+) MMI or PTU recipients group than in the CATD(-) group.

**Table 3:** Outcomes of RIT between the CATD(-)and CATD(+) groups

	CATD(-) group	CATD(+) group	*p*
**n**	15	15	-
Cured within 6 months from first RIT, n (%)	12 (80.0)	1 (6.7)	**< 0.001**
Cured within 12 months from first RIT, n (%)	12 (80.0)	4 (26.7)	**0.005**
Cured after 12 months from first RIT, n (%)	2 (13.3)	2 (13.3)	0.701
Required second RIT for cure, n (%)	1 (6.7)	9 (60.0)	**0.003**

Continuous variables are shown as medians (interquartile range). Categorical variables are presented as numbers (percentages)

Abbreviations: RIT, radioactive iodine treatment; CATD, RIT with concomitant antithyroid drugs

## Discussion

In this study, we report for the first time the efficacy of RIT with concomitant ATDs in Japanese patients with GD. Some studies reported a reduced cure rate in patients with GD when ATDs were continued during RIT [13], while others found no statistically significant difference [14, 15]. In this study, we performed propensity score matching to match each patient in the CATD(+) group with a patient in the CATD(-) group with the closest score. Nevertheless, a statistically significant difference in the cure rate within one year after RIT was found between the CATD(-) and CATD(+) groups (Table 3). The following factors may explain this difference in cure rates. First, there was a statistically significant difference in the daily ATD dose used before RIT between the two groups (Table 2). Although this is based on a single report, it has been suggested that a higher ATD dose before RIT, rather than the duration of ATD administration, is associated with and a lower cure rate of RIT [27], which may help explain our findings. Second, there were differences in patient background factors between the two groups. All patients in the CATD (+) group had complications from severe hyperthyroidism and experienced strong subjective symptoms. As a result, unlike the CATD (-) group, they may have required high doses of ATDs to maintain euthyroidism. As previously reported, severe hyperthyroidism is a factor known to reduce the efficacy of RIT [19, 22, 23, 25, 28]. In this study, at the first outpatient visit approximately 2 weeks after RIT, FT3 levels appeared to be slightly lower in the CATD(+) group. However, the difference was not statistically significant compared to the CATD(-) group (Fig. 2). Previous studies have reported mixed findings on whether RIT with concomitant ATDs can suppress or mitigate post-RIT increases in thyroid hormone levels. Some studies reported statistically significant suppression of FT4 in the continued-ATD group [14], while others found no difference between groups [15].

In conclusion, RIT with concomitant ATDs may result in a lower cure rate within 1 year compared with standard RIT involving temporary ATD discontinuation. However, it should be noted that all patients in the CATD(+) group in this study required high doses of ATDs and had complications from severe hyperthyroidism, including heart failure or thyroid storm, or experienced strong subjective symptoms. We suggest that further prospective studies be conducted to confirm the efficacy of radioactive iodine therapy combined with antithyroid drugs in countries with high iodine intake.

### Limitations

Our findings are based on retrospective analyses and may be limited by insufficient statistical power. Then, data were collected only from one hospital, which has an impact on the generalizability of our results. Furthermore, the 24-h RAIU values used in this study were estimated based on actual 3-h RAIU measurements and may not reflect true 24-h RAIU values. This study’s findings pertain specifically to patients with Graves’ disease who were receiving high-dose ATDs and had comorbidities such as heart failure or thyroid storm.

Abbreviations: RIT, radioactive iodine treatment; CATD, RIT with concomitant antithyroid drugs; FT4, free T4; FT3, free T3; ns, not significant.

## Supplementary Information

Below is the link to the electronic supplementary material.

Supplementary Material 1

Supplementary Material 2

Supplementary Material 3

Supplementary Material 4

Supplementary Material 5

## Data Availability

The study protocol and datasets are available from the corresponding author on request.

## Abbreviations

GD: Graves’ disease
RIT: Radioactive iodine therapy
ATDs: Antithyroid drugs
RAIU: Radioactive iodine uptake
NYHA: New York Heart Association
KI: Potassium iodide
MMI: Methimazole
PTU: Propylthiouracil
FT3: Serum free-T3
FT4: Serum free-T4
TW: Thyroid weight
