# Supplementary material for: Efficacy of radioactive iodine therapy with concomitant antithyroid drugs in Japanese patients with Graves’ disease: a retrospective observational study
Source: BMC Res Notes. 2025 Nov 12;18:476. doi: 10.1186/s13104-025-07558-9 (PMC12613672; doi:10.1186/s13104-025-07558-9)
Supplement: Supplementary file 2 — Supplementary Material 2 [file 13104_2025_7558_MOESM2_ESM.docx]

**Supplementary Table 1**. Characteristics of the entire CATD(-) group and the CATD(+) MMI recipients group after matching.

|  | CATD(-) group | CATD(+) group | *p* |
| --- | --- | --- | --- |
| **n** | 9 | 9 | - |
| Female, n (%) | 9 (100) | 8 (88.9) | 0.500 |
| Age (year) | 37 (27-51) | 40 (31-58) | 0.666 |
| Smokers, n (%) | 1 (11.1) | 0 (0) | 0.500 |
| TSH (µIU/mL) | 3.140 (0.555-5.9) | 0.466 (0-3.095) | 0.190 |
| FT4 (ng/dL) | 0.95 (0.91-1.46) | 1.04 (0.68-1.49) | 0.863 |
| FT3 (pg/mL) | 3.0 (2.9-4.1) | 4.1 (2.7-5.3) | 0.666 |
| TRAb (IU/L) | 1.3 (2.0-7.0) | 32.5 (10.0-39.3) | **0.031** |
| TW (g) | 63.3 (46.1-77.3) | 69.3 (47.4-81.3) | 0.489 |
| *MMI recipients, n (%) | 8 (88.9) | 9 (100) | 0.500 |
| *PTU recipients, n (%) | 0 (0) | 0 (0) | - |
| ATD dose (mg/day) | 200 (100-300) | 600 (450-700) | **<0.001** |
| KI (mg/day) | 0 (0-25) | 0 (0-50) | 0.730 |
| Iodine Restriction before RIT (day) | 7 (7-11) | 7 (7-9) | 0.297 |
| 3-h RAIU (%) | 53.1 (46.0-73.3) | 43.1 (32.5-52.1) | 0.050 |
| ^131^I dose (mCi) | 13 (13-13) | 13 (13-13) | 0.730 |
| **Patient’s Background Factors** |  |  |  |
| **Coexistence or history of heart failure, n (%) | 1 (11.1) | 3 (33.3) | 0.288 |
| History of thyroid storm, n (%) | 0 (0) | 2 (22.2) | 0.235 |
| ***Severe symptoms due to hyperthyroidism, n (%) | 0 (0) | 4 (44.4) | **0.041** |

Continuous variables are shown as medians (interquartile range). Categorical variables are presented as numbers (percentages).

* MMI and PTU recipients indicate the number of recipients of MMI or PTU before RIT, respectively.

** Coexistence or history of heart failure classified as New York Heart Association (NYHA) class II or higher.

*** Severe symptoms such as fatigue, palpitations, and shortness of breath that significantly impaired their ability to carry out normal daily activities owing to hyperthyroidism.

Abbreviations: RIT, radioactive iodine treatment; CATD, RIT with concomitant antithyroid drugs; FT4, free T4; FT3, free T3; TRAb, TSH receptor antibody; TW, thyroid weight; MMI, methimazole; PTU, propylthiouracil; ATD, antithyroid drug; KI, potassium iodide; RAIU, radioactive iodine uptake.
