# Supplementary material for: Efficacy of radioactive iodine therapy with concomitant antithyroid drugs in Japanese patients with Graves’ disease: a retrospective observational study
Source: BMC Res Notes. 2025 Nov 12;18:476. doi: 10.1186/s13104-025-07558-9 (PMC12613672; doi:10.1186/s13104-025-07558-9)
Supplement: Supplementary file 3 — Supplementary Material 3 [file 13104_2025_7558_MOESM3_ESM.docx]

**Supplementary Table 4**. Outcomes of RIT between the entire CATD(-) group and the CATD(+) PTU recipients group after matching.

|  | CATD(-) group | CATD(+) group | *p* |
| --- | --- | --- | --- |
| **n** | 5 | 5 | - |
| Cured within 6 months from first RIT, n (%) | 4 (80.0) | 0 (0) | **0.024** |
| Cured within 12 months from first RIT, n (%) | 4 (80.0) | 1 (20.0) | 0.103 |
| Cured after 12 months from first RIT, n (%) | 0 (0) | 0 (0) | - |
| Required second RIT for cure, n (%) | 1 (20.0) | 4 (80.0) | 0.103 |

Continuous variables are shown as medians (interquartile range). Categorical variables are presented as numbers (percentages).

Abbreviations: RIT, radioactive iodine treatment; CATD, RIT with concomitant antithyroid drugs; PTU, propylthiouracil.
