# Supplementary material for: Efficacy of radioactive iodine therapy with concomitant antithyroid drugs in Japanese patients with Graves’ disease: a retrospective observational study
Source: BMC Res Notes. 2025 Nov 12;18:476. doi: 10.1186/s13104-025-07558-9 (PMC12613672; doi:10.1186/s13104-025-07558-9)
Supplement: Supplementary file 4 — Supplementary Material 4 [file 13104_2025_7558_MOESM4_ESM.docx]

**Supplementary Table 2**. Characteristics of the entire CATD(-) group and the CATD(+) PTU recipients group after matching.

|  | CATD(-) group | CATD(+) group | *p* |
| --- | --- | --- | --- |
| **n** | 5 | 5 | - |
| Female, n (%) | 2 (40.0) | 1 (20.0) | 0.500 |
| Age (year) | 45 (37-54) | 40 (31-58) | 0.690 |
| Smokers, n (%) | 3 (60.0) | 2 (40.0) | 0.500 |
| TSH (µIU/mL) | 0.090 (0-2.438) | 0 (0-2.245) | 0.911 |
| FT4 (ng/dL) | 0.94 (0.82-2.42) | 1.09 (0.54-2.21) | 0.841 |
| FT3 (pg/mL) | 3.0 (2.7-6.5) | 2.4 (2.1-8.7) | 0.548 |
| TRAb (IU/L) | 3.0 (1.3-41.4) | 11.1 (4.9-84.5) | 0.222 |
| TW (g) | 43.9 (33.8-150.0) | 73.7 (40.0-77.4) | 0.548 |
| *MMI recipients, n (%) | 2 (66.7) | 0 (60.0) | 0.222 |
| *PTU recipients, n (%) | 2 (13.3) | 5 (40.0) | 0.083 |
| ATD dose (mg/day) | 300 (100-450) | 400 (150-750) | 0.548 |
| KI (mg/day) | 0 (0-75) | 100 (50-100) | 0.095 |
| Iodine Restriction before RIT (day) | 8 (7-11) | 8 (8-10) | 0.690 |
| 3-h RAIU (%) | 39.6 (24.5-72.6) | 42.4 (24.2-54.6) | 0.917 |
| ^131^I dose (mCi) | 13 (13-13) | 13 (13-13) | 1.00 |
| **Patient’s Background Factors** |  |  |  |
| **Coexistence or history of heart failure, n (%) | 0 (0) | 2 (40.0) | 0.222 |
| History of thyroid storm, n (%) | 0 (0) | 1 (20.0) | 0.500 |
| ***Severe symptoms due to hyperthyroidism, n (%) | 0 (0) | 2 (40.0) | 0.222 |

Continuous variables are shown as medians (interquartile range). Categorical variables are presented as numbers (percentages).

* MMI and PTU recipients indicate the number of recipients of MMI or PTU before RIT, respectively.

** Coexistence or history of heart failure classified as New York Heart Association (NYHA) class II or higher.

*** Severe symptoms such as fatigue, palpitations, and shortness of breath that significantly impaired their ability to carry out normal daily activities owing to hyperthyroidism.

Abbreviations: RIT, radioactive iodine treatment; CATD, RIT with concomitant antithyroid drugs; FT4, free T4; FT3, free T3; TRAb, TSH receptor antibody; TW, thyroid weight; MMI, methimazole; PTU, propylthiouracil; ATD, antithyroid drug; KI, potassium iodide; RAIU, radioactive iodine uptake.
